# Accelerometry is a valid method to distinguish between healthy and 6-OHDA-lesioned parkinsonian rats

**DOI:** 10.1038/s41598-025-17278-6

**Published:** 2025-08-29

**Authors:** Johannes Otto, Meike Statz, Hanna Weber, Maximilian Koschay, Maria Kober, Franz Plocksties, Dirk Timmermann, Christian Haubelt, Alexander Storch, Mareike Fauser, Florian Grützmacher, Sascha Spors

**Affiliations:** 1 Institute of Communications Engineering, University of Rostock, Albert-Einstein-Str. 26, Rostock, 18059 Germany; 2 Department of Neurology, University Medical Center Rostock, University of Rostock, Gehlsheimer Str. 20, Rostock, 18147 Germany; 3 Institute of Applied Microelectronics and Computer Engineering, University of Rostock, Albert-Einstein-Str. 26, Rostock, 18059 Germany; 4 German Centre for Neurodegenerative Diseases, Gehlsheimer Str. 20, Rostock, 18147 Germany

**Keywords:** 6-hydroxydopamine, Parkinson’s disease, Accelerometry, Biomarkers, MEMS accelerometer, Preclinical research, Parkinson's disease, Biomarkers

## Abstract

In Parkinson’s disease (PD), continuous sensor-based evaluation of motor symptom severity, e.g., using accelerometry, has become an emerging field of interest in clinical research. Continuous symptom monitoring would also be of interest in preclinical disease models; however, such devices are far less established in animal models, most likely due to additional requirements in size, energy consumption, and impairment-free attachment. In contrast, accelerometers manufactured in micro-electro-mechanical systems (MEMS) technology are promising sensor devices, which allow for space-saving and energy-efficient monitoring of movements. In the present study, we aim to extend the state of the art by establishing wireless accelerometer measurements as a simple and energy-efficient method to distinguish between healthy rats and the 6-hydroxydopamine (6-OHDA) PD animal model. Male Wistar-Han rats were assessed either three weeks after unilateral 6-OHDA or sham lesioning within their home cages with an extracorporeal accelerometer placed in a rodent backpack for 12 h during their active phase. The data was transmitted wirelessly to a computer, preprocessed, and a statistical analysis was performed to find differences between the datasets of 6-OHDA and sham-lesioned rats. The statistical analysis showed significant differences in the variances of the magnitude of the acceleration vectors between the two classes. In conclusion, accelerometry is a valid method to distinguish between 6-OHDA-lesioned rats with unilateral dopaminergic deficiency and their healthy counterparts. The presented method represents a first step towards automated symptom severity monitoring and provides a framework to expand the application to on-implant integrated accelerometers for continuous monitoring of symptom manifestations in rodent models of neurodegenerative diseases. Future studies are required to expand accelerometry to assess symptom severity to ultimately utilize it for preclinical research on adaptive therapies.

**Supplementary Information:**

The online version contains supplementary material available at 10.1038/s41598-025-17278-6.

## Introduction

Advanced Parkinson’s disease (PD) is still associated with a significant disease burden and an impaired quality of life, e.g., due to motor complications such as wearing-off, sudden-off, and dyskinesia^1–3^. Therefore, much effort has been put into the development of digital biomarkers from sensor-based devices to further optimise symptom control based on quasi-continuous assessments of symptom severities^4–9^. In addition, such devices might aid with diagnosis and have been shown to even identify prodromal PD years ahead of clinical diagnosis^10,11^. However, when thinking about advancements in adaptive PD therapies, e.g., adaptive deep brain stimulation (aDBS^12,13^;) or closed-loop systems for continuous drug application (reviewed in^14^), there is still a need for preclinical studies in animal models of the disease to further advance these therapeutic approaches. While there have been first attempts in monitoring acceleration in healthy animals^15,16^ or in PD mouse models^17^, their head-mounted approaches, especially the wired approaches in^15,16^, impair free movement of the animals and therefore can distort the results of motion sensing. In order to address this limitation, a possible monitoring solution could be based on wireless sensors worn as backpacks or even implantable wireless sensors^18^. However, continuous symptom monitoring with wireless sensors faces special challenges. Due to its mobile character, the sensor system is required to be battery-driven. In addition, the wireless sensor system, as well as its battery, have to be small enough to allow unimpeded movement of the animal. This, however, causes an even more constrained energy budget of the wireless sensor system, which necessitates ultra-low-power techniques for the sensing and wireless transmission of sensor signals^19^. In order to tackle these challenges, micro-electro-mechanical systems (MEMS) based accelerometers^20^ pose a promising alternative to capture motor symptoms in Parkinsonian rats in an energy efficient way. This stems from their small device size of, e.g., less than 4 mm$^3$^21^, which allows integration into miniature sensor devices and even into implantable devices like STELLA+^18^, on the one hand, and due to their low current consumption of as low as 850 nA^21^, on the other hand. Consequently, low-power wireless standards, e.g., bluetooth low energy (BLE), need to be utilized in an energy efficient way, in order to allow for longer monitoring periods. In addition to the constrained energy budget, monitoring of small animals, e.g., rodents, by accelerometers poses further challenges. Due to the small body size, movements are more subtle. This introduces additional complexities in identifying corresponding movements in accelerometer signals which are inherently subject to noise. Compared to studies that involve larger animals^22–25^, the movement of rodents create smaller amplitudes which increases the significance of noise in accelerometer signals.

Ultimately, despite the aforementioned challenges, preclinical monitoring with on-implant accelerometers could represent a method for the automated identification of motor symptom severity of 6-hydroxydopamine (6-OHDA)-lesioned Parkinsonian rats with a very low restriction of movement and behavior. In order to advance the current research, it is crucial to evaluate in a first step, whether accelerometry is capable of capturing movements that distinguish healthy from 6-OHDA-lesioned Parkinsonian rats. For this purpose, we used wireless sensor nodes equipped with a MEMS accelerometer and a BLE wireless transceiver module, carried in a rodent backpack for continuous movement monitoring of up to 24 h.

## Results

**Fig. 1:**
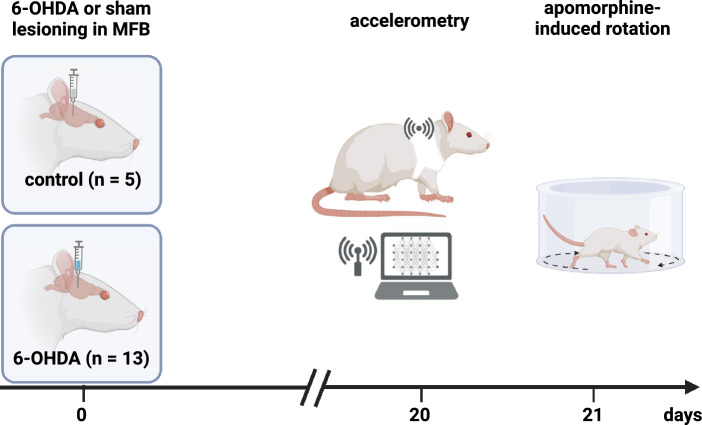
Experimental design. Animals received either sham (healthy control animals) or 6-hydroxydopamine (6-OHDA) injections into the right median forebrain bundle (MFB). After 20 days, animals underwent accelerometer experiments overnight in their active phase (lights off) and were subjected to apomorphine-induced rotational testing to quantify the dopaminergic deficit the next day. (Created in BioRender. Statz, M. (2025) https://BioRender.com/o44t967).

Three-dimensional acceleration signals of $D_{all}=18$ animals, of which 5 were sham- and 13 6-OHDA-lesioned, were recorded over a period of 12 hours. A single continuous dataset was recorded per animal. Afterwards apomorphine-induced rotation was used to quantify the extent of the PD phenotype of each subject: while sham animals displayed a mean of 0.16 ± 0.20 rpm, 6-OHDA Parkinsonian animals showed 7.18 ± 0.30 rpm (p < 0.0001, unpaired t-test with Welch´s correction; see Table 1), indicating a significant dopaminergic deficit three weeks after initial lesioning (data already published in^26^). The corresponding experimental design and schedule are visualized in Fig. 1. The magnitudes *m* of the three-dimensional acceleration vectors were calculated and used for all further evaluation. The magnitude signals were then inspected by calculating several statistical measures, as described in the following sections, in order to identify differences between the two classes. During the investigation, 3 datasets (all belonging to the PD class) were classified as outliers and removed from the datasets, bringing the final dataset number to $D_{final}=15$. Each dataset $M_d = (m_{d,1}, \dots , m_{d,i}, \dots , m_{d,I})$, where $1 \le d \le D_{final}$ and *i* denoting the individual samples with $1 \le i \le I$, has a length of $I=1080000$ samples. This equals 12 h of recorded data, considering that one timestep $i \rightarrow i+1$ equals 0.04 s for the used sampling rate of $f_s = 25 Hz$. All individual datasets are combined into the data-matrix $\varvec{M} = (M_1, \dots , M_d, \dots , M_D)^T$ of $D \times I$ dimensions.

**Fig. 2:**
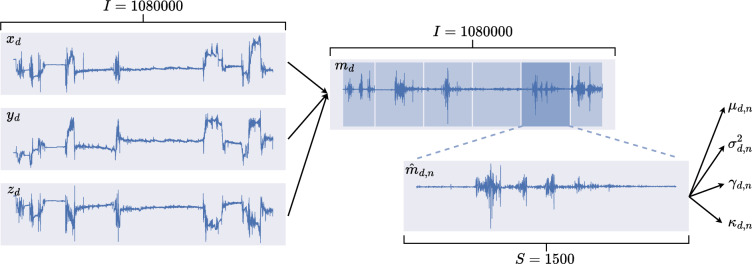
Overview of the signal processing for the evaluation of the segmental statistics. The magnitude $m_d$ is calculated from the three acceleration axis $x_d, y_d, z_d$, each of length $I=1080000$ per dataset *d* (Note that the actual signal presented here is trimmed to only 8000 samples for better visualization). $m_d$ is then segmented into $N=720$ segments $m_{d,n}$ of length $S=1500$. From these segments the first four statistical moments are computed.

**Fig. 3:**
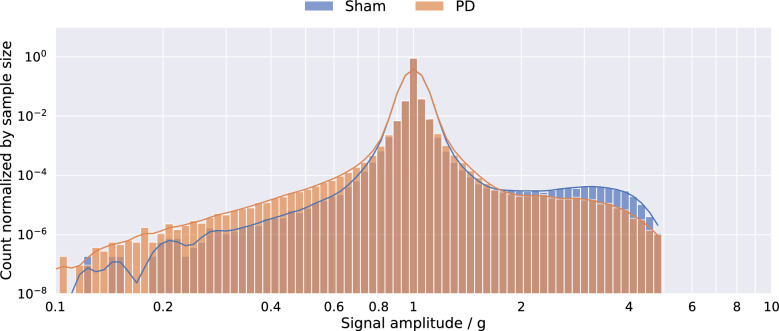
Histogram of amplitudes of sham and Parkinson’s disease (PD) model datasets.

**Fig. 4:**
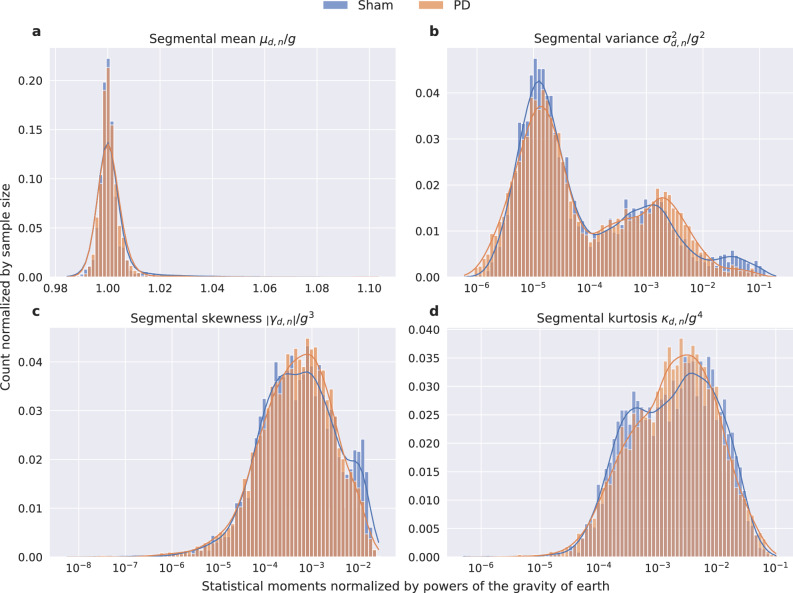
Histograms of segmental mean $\mu _{d,n}$, variance $\sigma ^2_{d,n}$, skewness $\gamma _{d,n}$, and kurtosis $\kappa _{d,n}$. All values are normalized by powers of g.

The histogram of the acceleration magnitudes of the sham ($d\le 5$) and PD datasets ($d>5$), which is shown in Fig. 3, indicates differences between the two classes to be mostly in the form of higher relative occurrences of magnitudes above $1~g = 9.81 \frac{m}{s^2}$ for the sham class. Note that the configured range of $\pm ~4~g$ applies to each individual axis of the acceleration sensor. As a result, the magnitude of the acceleration vector may be higher, which can be observed in Figure 3. In order to assess the differences between the two classes, segmental statistics were calculated, i.e., the data was segmented and for each individual segment the mean $\mu _{d}$, variance $\sigma ^2_{d}$, skewness $\gamma _{d}$, and kurtosis $\kappa _{d}$ were computed. An overview of the signal processing is given in Fig. 2. To gain insights into the class-related differences between these statistical distributions, the statistics of the segments were pooled for each class. Then, superimposed histograms of both classes were plotted for all four statistical moments. These are presented in Fig. 4. Differences between classes in the histogram of the segmental means (Fig. 4a) are small with most means at approximately 1 *g*. Differences in the histogram of segmental variances (Fig. 4b) mostly occur above 0.01, where the sham group’s distribution is more present. The distribution of segmental skewness (Fig. 4c) displays a peak above 0.005 for sham datasets, deviating from the distribution of PD segments. The same deviation can be seen in the distribution of segmental kurtosis (Fig. 4d), although to a much lesser extent. In addition to the evaluation of class-related differences, these histograms are also available with the individual animals distinguished by color in Supplementary Figure S 1.

**Fig. 5:**
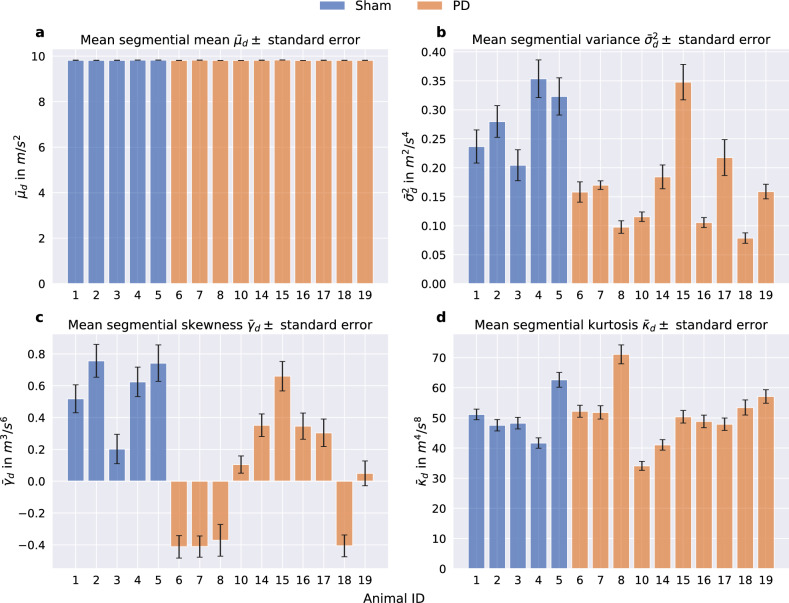
Means of segmental mean $\bar{\mu }_{d}$, variance $\bar{\sigma }^2_{d}$, skewness $\bar{\gamma }_{d}$, and kurtosis $\bar{\kappa }_{d}$. The black error bars depict the standard error.

The results of the mean segmental statistics per dataset $\hat{M}_{d}$ are shown in Fig. 5. The mean segmental mean $\bar{\mu }_{d,n}$ (Fig. 5a) equals approximately 1 *g* for all individuals, with a decrease of $0.04~\%$ from $9.822~\frac{m}{s^2}$ for sham to $9.818~\frac{m}{s^2}$ for 6-OHDA-lesioned animals. The mean segmental variance $\bar{\sigma }^2_{d,n}$ (Fig. 5b) shows differences between sham and PD animals. The majority of variances in the sham group seem to be higher than for 6-OHDA animals. The mean variance across the two classes is decreased by $41.5~\%$ from $0.279~\frac{m^2}{s^4}$ for sham to $0.163~\frac{m^2}{s^4}$ for PD animals. Similar results can be seen for the mean segmental skewness $\bar{\gamma }_{d,n}$ (Fig. 5c), with a decrease of $39.9~\%$ from $0.569~\frac{m^3}{s^6}$ to $0.342~\frac{m^3}{s^6}$. The mean segmental kurtosis $\bar{\kappa }_{d,n}$ (Fig. 5d) shows almost no difference, with a very small increase of $1.07~\%$ between the sham group with $50.258~\frac{m^4}{s^8}$, and 6-OHDA-lesioned animals with $50.798~\frac{m^4}{s^8}$. In addition to the evaluation on acceleration magnitudes as described in this section, histograms were furthermore calculated for each individual axis x, y, and z of the acceleration sensor signal, to its first integration (velocity), its second integration (position), and for their combination as spherical coordinates $\phi$ and $\theta$ in order to reveal possible asymmetries or directional bias in movements due to the unilateral dopaminergic deficiency. These evaluations did not lead to statistically relevant results. However, for the sake of completeness, their corresponding histograms are available as supplementary information in Supplementary Figure S 2, S 3, and S 4.

Three hypothesis tests have been performed for the null hypothesis $H_{0, \bar{\sigma }^2_{d}}$, that the mean segmental variances for sham and PD datasets have the same underlying distribution. Although the group sizes are limited due to technical and regulatory reasons in our study, the hypothesis tests still provide valuable insights since the group sizes of our study are in line with similar reports in the literature^15,17^ as well as the fact that the utilized statistical tests take groups sizes inherently into account. In order to provide conservative measures, three statistical tests have been performed and their p-values have been adapted with Holm-Bonferroni adjustment^27^ to further correct for skewed results due to multiple testing. Both their Holm-Bonferroni-adjusted p-values $\tilde{p}$ and the un-adjusted p-values *p* are reported. The results are sorted according to the Holm-Bonferroni algorithm.

The results are $\tilde{p} = 0.038$ ($p = 0.013$) for the Mann-Whitney-U test, $\tilde{p} = 0.035$ ($p = 0.018$) for the Baumgartner-Weiß-Schindler test, and $\tilde{p} = 0.019$ ($p = 0.019$) for the Kolmogorov-Smirnov test. Considering the significance level $\alpha = 0.05$, all tests are declared significant. The same tests have been performed for the null hypothesis $H_{0, \bar{\gamma }_{d}}$, that the mean segmental absolute skewness for sham and PD datasets has the same underlying distribution. The results are, $\tilde{p} = 0.058$ ($p = 0.019$) for the Mann-Whitney-U test, $\tilde{p} = 0.063$ ($p = 0.032$) for the Baumgartner-Weiß-Schindler test, and $\tilde{p} = 0.061$ ($p = 0.061$) for the Kolmogorov-Smirnov test. In this case all Holm-Bonferroni corrected p-values are above $\alpha$, therefore $H_{0, \bar{\gamma }_{d}}$ cannot be rejected and non of the tests regarding the segmental skewness are declared significant. As a result, among all features that have been analyzed in this study, only the variance of the magnitudes of the acceleration vectors is a valid feature to distinguish between healthy and 6-OHDA-lesioned rats. In order to test whether the variance of magnitudes indicates the severity of 6-OHDA-lesioning, we analyzed their correlation w.r.t. the apomorphine-induced rotations, which can be considered a measure of the degree of dopaminergic degeneration. The analyses showed no significant correlation. Corresponding data is depicted in Supplementary Figure S 5. As a result, the variance of the acceleration magnitudes indicates differences between the two classes sham and 6-OHDA-lesioned rats. However, the variance does not represent a biomarker for the severity of the lesioning and further research in this direction, potentially with higher sampling frequencies, additional sensor modalities, e.g., magnetometers, and more sophisticated signal processing, is necessary.

## Discussion

This study utilizes, for the first time, acceleration measurements in a preclinical PD animal model to distinguish Parkinsonian rats from their healthy counterparts. It is important to note that, while for larger animals accelerometry is a state of the art method for behavioral analysis^22^, the movement of smaller animal models however, i.e., rodents, have a less pronounced amplitude in accelerometer signals w.r.t. to the inherently present noise level of sensors. As a result, differences between healthy and Parkinsonian rats are unequally harder to identify, which necessitates further research. So far, acceleration measurements have been carried out primarily in healthy small animal models in the literature (e.g., in^28^) and less frequently in rodent PD models, the literature of which is discussed in the following. Regarding PD animal models, a very recent study reported quantification of dyskinesia in a PD mouse model using wireless inertial measurement unit (IMU) sensors with a recording time of 4 h^17^. However, in their study, sensors were head-mounted, which enables simultaneous recording of striatal activity, but might significantly impair free movements. Furthermore, the IMU integrated gyroscopes were utilized in addition to accelerometers, which significantly increases the energy consumption of the wireless sensor device, due to a higher energy consumption of gyroscopes in comparison to accelerometers^29,30^. Another study used external accelerometers placed on head caps to measure tremor induced by specifically patterned deep brain stimulation in healthy rats^31^. In the present study, only a single accelerometer is used, which allowed for a much longer observation time of up to 24 h. Furthermore, the present study shows that accelerometers on their own are sufficient to distinguish Parkinsonian rats from their healthy counterparts.

Other existing reports in healthy animals used head-mounted, wired accelerometers^15,16^, which prevents unimpeded movement of the animals even more so. The backpack-worn accelerometers in the present study should reduce movement constraints, but will not completely avoid them. In the future, such limitations could be fully overcome by implantable devices, such as the recently published STELLA+ device^18^, which has been developed as a preclinical neurostimulator, but also integrates a BMA400 MEMS accelerometer, which allows for accelerometer measurements in freely moving animals.

This study is limited in terms of its small animal numbers due to restrictions of the complex experimental setup, i.e., limited number of devices due to interference during data transmission with chosen BLE transceivers and an imbalance between the group sizes. Nevertheless, the group sizes used in this study are in line with similar reports in the literature^15,17^. Furthermore, the utilized statistical tests take group sizes inherently into account. Another limitation of this study arises from the inclusion of animals of one sex, which restricts the generalizability and reproducibility of the findings. However, previous studies have indeed demonstrated differences in the extent of dopaminergic degeneration as well as in the persistence of motor impairments between male and female 6-OHDA-treated rats^32,33^. In our present study, accelerometry in healthy control animals was only performed on the first day of the study (see Table 1 for details), which might influence the data regarding external confounders (e.g., noise, vibrations).

**Table 1:** Overview of *in vivo* data acquisition.

Day	Animal ID	Condition	Sensor ID	Apomorphine-induced rotation in rpm
1	1	Control	S3	−0.01
	2	Control	S4	−0.14
	3	Control	S1	0.99
	4	Control	S2	0.27
	5	Control	S5	−0.32
	6	6-OHDA	S6	9.00
	7^*^	6-OHDA	S7/S7	5.71
	8	6-OHDA	S8	7.56
2	9	6-OHDA	S5	5.48
	10	6-OHDA	S6	7.08
	11^§^	6-OHDA	S1	9.40
	12	6-OHDA	S2	6.58
	13^+^	6-OHDA	S3	−0.16
	14	6-OHDA	S4	7.76
3	15	6-OHDA	S1	7.58
	16	6-OHDA	S2	7.69
	17	6-OHDA	S3	7.55
	18	6-OHDA	S4	6.03
	19	6-OHDA	S6	7.21
	20	6-OHDA	S8	5.82

Sensor allocation by date and animal number. ^*^Data from animal 7 on day 1 was excluded due to problems with the animal’s backpack and repeated on day 2. ^§^The dataset from animal 11 was omitted due to sensor failure during data acquisition. ^+^Data from animal 13 was excluded due to insufficient lesioning.

The reduced motor activity of 6-OHDA-lesioned animals, which might be interpreted as bradykinesia, one of the cardinal motor symptoms of PD^34^, can be observed in the histogram of the magnitude of the raw accelerometer data. As expected, the sham data’s distribution is more pronounced at higher accelerations than the PD data. The distribution of statistical moments of the one-minute segments shows differences between the classes as well. However, these statistical differences are not sufficient to classify each segment on its own. The means of the variances over the whole 12-hour period, however, are a capable classifier, as was proven by hypothesis testing.

## Methods

### Animals

All procedures were approved by responsible authorities (Landesamt für Landwirtschaft, Lebensmittelsicherheit und Fischerei, Mecklenburg-Vorpommern, Germany; reference number 7331.3-1.011/21) and performed in accordance with the relevant guidelines and regulations (ARRIVE guidelines and EU Directive 2010/63/EU). We used 20 male Wistar-Han rats (260-280 g at the time of arrival; Charles River Laboratories, Germany) of which 18 were included into final analyses (two drop-outs due to device failures or insufficient lesioning; as detailed in Figure 1 and Table 1). All animals were housed in pairs in a 12 h-light-dark cycle and had *ad libitum* access to food and water. A subset of rats (n = 15) underwent right-sided unilateral 6-OHDA lesioning to generate a reliable dopaminergic degeneration as described previously in detail^26,35^. Briefly, rats were placed in a stereotaxic frame (Stoelting Neuroscience, Ireland) anesthetized with isoflurane (5% in 1 l/min O_2_ for 1 min, reduced to 2-2.5% during procedures) followed by weight-adapted ketamine/xylazine administration (1.4 ml/kg bodyweight of 25 mg/ml ketamine (Pfizer, Germany) and 20 mg/ml xylazine (Rompun, Bayer Healthcare, Germany)) and injected with a total of 4 $\upmu$l 6-OHDA (6 $\upmu$g/$\upmu$l in 0.9% NaCl with 0.02% ascorbic acid; Sigma-Aldrich, UK) into the right median forebrain bundle (MFB) at the following coordinates: anterior-posterior (AP) −2.3 mm; medial-lateral (ML) −1.5 mm, dorsal-ventral (DV) −9.0 mm relative to bregma and dura according to the rat brain atlas^36^. Control animals (n = 5) underwent the same procedure except that they were injected with 4 $\upmu$l 0.9% NaCl with 0.02% ascorbic acid. Three weeks after lesioning, animals were singularized and equipped with a rodent backpack overnight, in which the wireless sensor nodes were placed. In order to reduce stress and detect animals’ natural behavior, backpacks were already pulled on several hours before data acquisition, which started at 7:45 p.m. and was continued until 7:45 a.m. with lights switched off (active phase of the animals). Successful 6-OHDA lesioning was quantified by apomorphine-induced rotational behavior three weeks after lesioning. We used 0.25 mg/kg body weight apomorphine (0.2 mg/ml in 0.9% NaCl; Teclapharm, Germany) and quantified rotational behavior for a total of 50 min (results have already been published in^26^).

### Accelerometry

Eight custom wireless sensor nodes equipped with a BHI160 sensor hub^37^ (Bosch Sensortec GmbH, Germany) were used. Three-axis acceleration measurements were acquired at a rate of 25 Hz with 16 bit resolution over a range of $\pm ~4~g$ for each axis. The sensor nodes were further equipped with a DA14583 system-on-chip (Dialog Semiconductor plc., UK) with an integrated BLE radio transceiver and baseband processor that was used to transmit acceleration data wirelessly to a data aggregating device. A CR1225 lithium coin cell with 3 V supply voltage and 50 mAh capacity was used to power the sensor nodes. This setup allowed a continuous acquisition of acceleration data up to 24 h. A Raspberry Pi 4^38^ single-board computer was used as data aggregating device, allowing acquisition of acceleration data from eight sensor nodes simultaneously via BLE with the specified settings. The nodes were housed in a 3D printed encapsulation using polylactic acid and placed in a rodent backpack. Sensors were used on three consecutive nights as detailed in Table 1. In addition, animals were continuously video-monitored with a 5 mega-pixel infrared camera with integrated infrared spotlight. The camera was connected to the above-mentioned Rasberry Pi 4, which recorded and stored the videos with 1080p in horizontal orientation in H.264 format on an external solid-state-drive.

### Data analyses

#### Preprocessing

In order to reduce the dimensionality of the data, the magnitude $m_{d,i}$ of the acceleration vector was computed from the raw three-dimensional acceleration measurements

1 $$\begin{aligned} m_{d,i} = \sqrt{x_{d,i}^2+y_{d,i}^2+z_{d,i}^2} \end{aligned}$$

with $1 \le d \le D_{all}$ being the animal ID, *i* denotes the discrete time index and $x_{d,i}$, $y_{d,i}$, $z_{d,i}$ the raw samples of the acceleration measurements of the three axes.

The datasets $M_d$ are then trimmed to a consistent length of $I=1080000$ or 12 h, respectively, starting at a consistent time of 7:45 p.m. During this time, artificial lighting was switched off and daylight was blocked out. This 12 h period of darkness is considered the active phase of the animals.

**Fig. 6:**
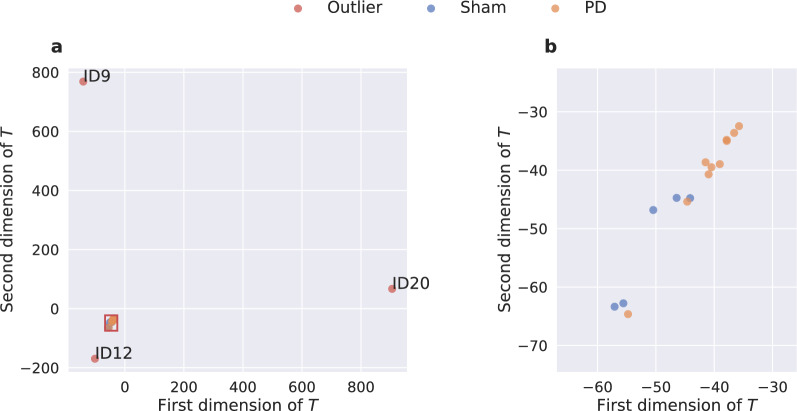
Two-dimensional latent space representation of the complete datasets. The dataset dimensions were reduced through principle component analysis. (**b**) is a zoomed in version of (**a**) with the magnified region indicated by the red rectangle in (**a**). The outliers with the ID’s 9, 12, and 20 are cropped out.

#### Quality measures

To be able to visualize differences between the datasets, a principal component analysis (PCA) was performed. The PCA is a tool to reduce the dimensionality of a dataset by projecting the data onto a new coordinate system. The new system’s axes are chosen such that the variance across the first axis is maximized. The variance across the subsequent axes is maximized as well, but under the condition that the axis is orthogonal to the other axes. The $D_{all} \times I$ matrix $\varvec{M}$ of all datasets $M_{d}$ is factorized through singular value decomposition into:

2 $$\begin{aligned} \varvec{M} = U \Sigma V^T, \end{aligned}$$

where *U* is a $D_{all} \times D_{all}$ and *V* is an $I \times I$ matrix. Both matrices are unitary. $V^T$ is the transpose of *V*. The $D_{all} \times I$ diagonal matrix $\Sigma$ contains the singular values $\sigma _i$ of $\varvec{M}$ on its diagonal. The transformation to the new coordinate system can be described by:

3 $$\begin{aligned} \varvec{T} = \varvec{M}V = U \Sigma . \end{aligned}$$

The PCA was computed through the sklearn.decomposition PCA function^39^. The index *I* of $\varvec{T}$ was cut off after the second dimension, giving a two-dimensional latent space representation in the form of a $D_{all} \times 2$ matrix. A scatterplot of this matrix can be seen in Fig. 6a. The three datasets with the IDs 9, 12, and 20 are noticeably different. We consider these as outliers. A zoomed-in version, with the outliers cropped out, is provided in Fig. 6b. The remaining datasets are distributed quite compactly, even some amount of clustering for the two classes sham and PD can be observed.

Based on the median-absolute-deviation (MAD), R. Wilcox proposed a MAD-median rule^40^. It was used, in addition to the PCA, to test for outliers. It was calculated separately for sham and PD datasets for the overall variance per dataset $\sigma ^2_{d}$ in the following way:

4 $$\begin{aligned} \frac{|\sigma ^2_{d}-median(\sigma ^2_{d})|}{MAD(\sigma ^2_{d})/0.6745}> K \end{aligned}$$

with $K = \sqrt{\chi ^2_{0.975,1}} \approx 2.24$ as the square root of the 0.975 quantile of a chi-squared distribution with one degree of freedom. Table 2 lists the scores for the datasets which are above the threshold *K*. The animals with IDs 9, 12, and 20 stand out again, with scores well above the threshold $K = 2.24$. This, combined with the PCA results, led to the decision to exclude these datasets from the database.

**Table 2:** Scores of median-absolute-deviation (MAD) rule for datasets with scores above the threshold $K=2.24$.

Condition	Animal ID	MAD-median score
PD	9	4.812
	12	4.036
	20	7.038

#### Analysis

In order to gain insight into the differences in terms of the statistical properties of the acceleration magnitudes, histograms of the two classes’ magnitude values $m_{d,i}$ were computed. The magnitude values $m_{d,i}$ were normalized by the gravity of Earth *g*:

5 $$\begin{aligned} \tilde{m}_{d,i} = \frac{m_{d,i}}{g}. \end{aligned}$$

To compute short-term statistics, the magnitude samples were further split temporally into segments of equal length $S = 1500$ (one minute of data) with no overlap

6 $$\begin{aligned} \hat{m}_{d,n} = (m_{d,(n-1)S + 1}, \dots , m_{d,n S}) = (m_{d,n,1}, \dots , m_{d,n,s}, \dots , m_{d,n,S}) \end{aligned}$$

where $1 \le n \le N$ denotes the segment index with the total number of segments per dataset $N = 720$ and *s* the discrete time index within each segment. The matrix

7 $$\begin{aligned} \hat{M}_d = (\hat{m}_{d,1}, \dots , \hat{m}_{d,N}) \end{aligned}$$

can then be defined, that includes all segments $\hat{m}_{d,n}$ generated from one dataset $M_d$. Now

8 $$\begin{aligned} mean(\hat{m}_{d,n}) = \mu _{d,n} = \frac{1}{S} \sum _{s=1}^S m_s, \end{aligned}$$

9 $$\begin{aligned} variance(\hat{m}_{d,n}) = \sigma ^2_{d,n} = \frac{1}{S-1} \sum ^S_{s = 1} (m_s - \mu _{d,n})^2, \end{aligned}$$

10 $$\begin{aligned} skewness(\hat{m}_{d,n}) = \gamma _{d,n} = \frac{\frac{1}{S} \sum ^S_{s = 1} (m_s - \mu _{d,n})^3}{\sqrt{\sigma ^2_{d,n}}^3}, \end{aligned}$$

and

11 $$\begin{aligned} kurtosis(\hat{m}_{d,n}) = \kappa _{d,n} = \frac{\frac{1}{S} \sum ^S_{s = 1} (m_s - \mu _{d,n})^4}{\sqrt{\sigma ^2_{d,n}}^4}, \end{aligned}$$

with $m_{d,n,s}$ shortened to $m_s$, were calculated for all segments $\hat{m}_{d,n}$. The segmental means, variances, skewnesses, and kurtosises of all segments were then normalized by the gravity of Earth *g*, whereby the order of the statistical measure was considered in the normalization:

12 $$\begin{aligned} \tilde{\mu }_{d,n} = \frac{\mu _{d,n}}{g},\ \tilde{\sigma }^2_{d,n} = \frac{\sigma ^2_{d,n}}{g^2},\ \tilde{\gamma }_{d,n} = \frac{\left| \gamma _{d,n}\right| }{g^3},\ and\ \tilde{\kappa }_{d,n} = \frac{\kappa _{d,n}}{g^4}. \end{aligned}$$

To amplify differences in the distributions at higher values, the histograms were generated using logarithmic bins. The x-axis was scaled accordingly. To avoid negative values in the logarithm, only the absolute values of skewness were used. The histograms are illustrated in Fig. 4. To further aggregate the statistics, the means of the segmental statistics per individual animal *d*

13 $$\begin{aligned} mean(\mu _{d,n}) = \bar{\mu }_{d},\ mean(\sigma ^2_{d,n}) = \bar{\sigma }^2_{d},\ mean(\gamma _{d,n}) = \bar{\gamma }_{d},\ and\ mean(\kappa _{d,n}) = \bar{\kappa }_{d} \end{aligned}$$

over all *N* segments were calculated. The mean segmental statistics are illustrated in Fig. 5.

Hypothesis tests were performed to test the null hypothesis $H_{0, \bar{\sigma }^2_{d}}$ that the population underlying the mean variances $\bar{\sigma }^2_{d}$ of the sham datasets is the same as the one underlying the PD datasets. The same tests have been performed for the null hypothesis $H_{0, \bar{\gamma }_{d}}$ that the population underlying the mean skewness $\bar{\gamma }_{d}$ is the same for both classes. The performed tests include Kolmogorov-Smirnov^41^, Mann-Whitney-U^42^, and Baumgartner-Weiß-Schindler^43^. All of these tests are non-parametric tests that do not assume normality for the data distributions. The tests have been performed excluding the outliers. The corresponding scipy.stats functions^44^ were used for calculation. The test results are compared against the significance level $\alpha = 0.05$. Because multiple hypothesis tests are performed, the family-wise error rate should be controlled in some way. To this end, we chose the Holm-Bonferroni correction^27^. The reported p-values are sorted from lowest to highest, and then adjusted with

14 $$\begin{aligned} \tilde{p_i} = p_i(N_{HT}+1-i), \end{aligned}$$

where $N_{HT} = 3$ is the number of hypothesis tests performed, and $1\le i \le N_{HT}$ the index of the sorted p-values. The sorted and adjusted p-values $\tilde{p_i}$ are then compared against $\alpha$ in order of their index *i*. If $\tilde{p_i}>\alpha$, the test is not declared significant and all subsequent tests are declared non-significant as well, even if $\tilde{p_{i+1}}<\alpha$.

## Conclusions

In our study, we utilized accelerometer measurements from wireless, rodent backpack-worn sensor nodes in order to distinguish between healthy and diseased rats in the 6-OHDA animal model of Parkinson’s disease. Our statistical analysis showed significant differences in the variances of the magnitude of the acceleration vectors for each dataset between healthy and diseased animals. As a conclusion, accelerometry is a valid method to distinguish between 6-OHDA-lesioned rats with unilateral dopaminergic deficiency and their healthy counterparts. Furthermore, our findings w.r.t. a correlation between the acceleration variance and severity of lesioning (apomorphine-induced rotations) show that while movement differences can be captured with accelerometer signals, the variance of magnitude does not reflect the final biomarker that indicates severity of the disease. As a conclusion, further research with potentially higher sampling frequencies and more sophisticated statistical or signal processing based data analysis approaches are necessary in order to identify relevant signal features that indicate symptom severity.

In future studies, the incorporation of additional low-power sensor data, e.g., magnetometers, could improve these results by allowing the reconstruction of the sensor’s orientation relative to earth’s coordinate system. Moreover, higher sampling rates and increased animal numbers in combination with machine learning approaches may significantly shorten the segment length needed for reliable classification.

Furthermore, our findings show promising potential for the long-term accelerometer monitoring with the recently published STELLA+ device^18^, which also integrates a MEMS based accelerometer and a BLE transceiver module.

Finally, we plan on applying this approach to other animal models of PD, including rodent models of $\alpha$-synucleinopathies, which better mimic the human disease and present with different motor symptom severities^45,46^. These findings may contribute to advancements in adaptive therapies in PD.

## Electronic supplementary material

Below is the link to the electronic supplementary material.

Supplementary Material 1

## Data Availability

The datasets generated and analyzed during the presented study consist of $\approx$ 350 GB of video material and $\approx$ 2.3 GB of raw acceleration data and will be made available by the corresponding author upon reasonable request.
