# Supplementary material for: Accelerometry is a valid method to distinguish between healthy and 6-OHDA-lesioned parkinsonian rats
Source: Sci Rep. 2025 Aug 29;15:31883. doi: 10.1038/s41598-025-17278-6 (PMC12397390; doi:10.1038/s41598-025-17278-6)
Supplement: Supplementary file 1 — Supplementary Material 1 [file 41598_2025_17278_MOESM1_ESM.pdf]

Accelerometry is a valid method to distinguish between healthy and 6-OHDA-lesioned parkinsonian rats

Johannes Otto[1,+,*], Meike Statz[2,+], Hanna Weber[2], Maximilian Koschay[3], Maria Kober[2], Franz Plocksties[3], Dirk Timmermann[3], Christian Haubelt[3], Alexander Storch[2,4], Mareike Fauser[2], Florian Grützmacher[3,+], and Sascha Spors[1,+]

[1]Institute of Communications Engineering, University of Rostock, Albert-Einstein-Str. 26, 18059 Rostock, Germany
[2]Department of Neurology, University Medical Center Rostock, University of Rostock, Gehlsheimer Str. 20, 18147 Rostock, Germany
[3]Institute of Applied Microelectronics and Computer Engineering, University of Rostock, Albert-Einstein-Str. 26, 18059 Rostock, Germany
[4]German Centre for Neurodegenerative Diseases, Gehlsheimer Str. 20, 18147 Rostock, Germany
[*]johannes.otto@uni-rostock.de
[+]these authors contributed equally to this work and share first and last authorship, respectively

Supplemental Material

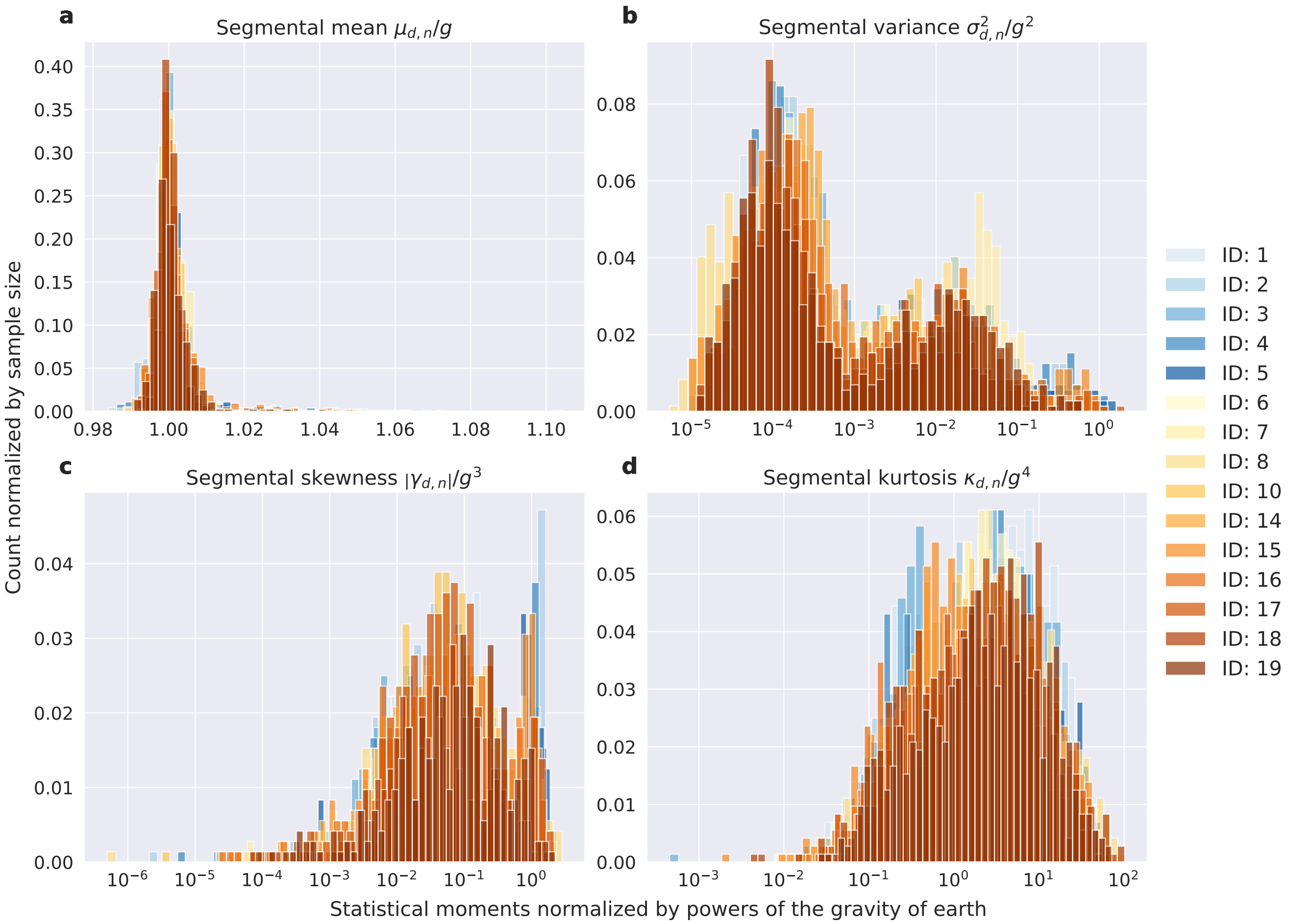

Supplementary Figure S 1. Histograms of segmental mean $\mu_{d,n}$, variance $\sigma^2_{d,n}$, skewness $\gamma_{d,n}$, and kurtosis $\kappa_{d,n}$. All values are normalized by powers of g. The individuals are separated by colors, where the sham class is colored in shades of blue and the PD class in shades of red.

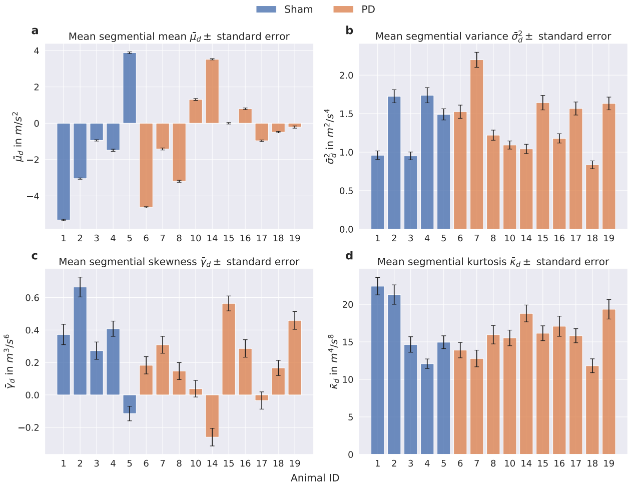

(a) x-axis

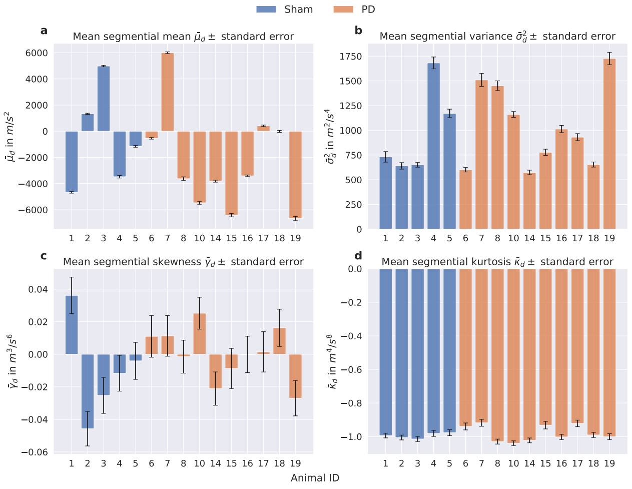

(b) x-axis, integrated once (velocity)

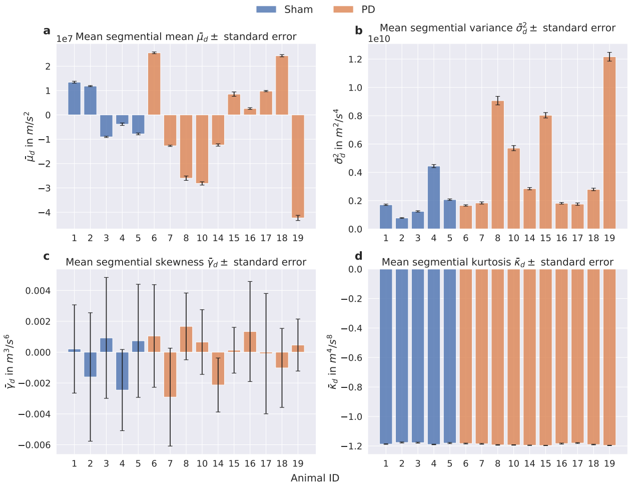

(c) x-axis, integrated twice (position)

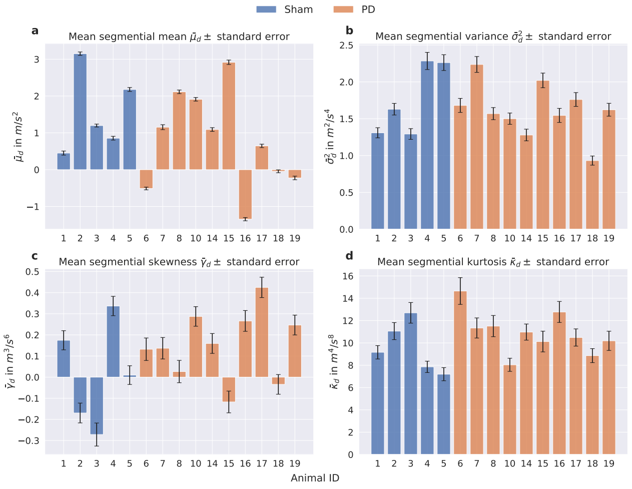

(d) y-axis

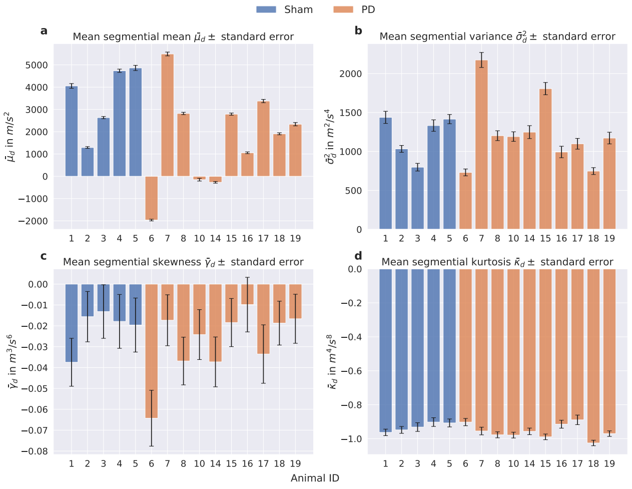

(e) y-axis, integrated once (velocity)

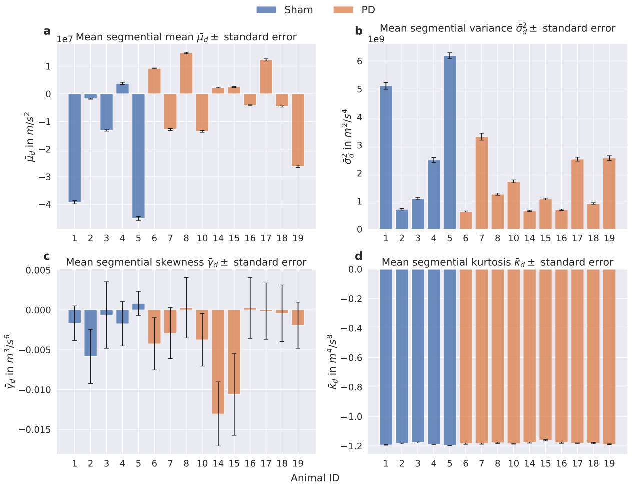

(f) y-axis, integrated twice (postition)

Supplementary Figure S 2. Means of segmental mean $\bar{\mu}_d$, variance $\bar{\sigma}_d^2$, skewness $\bar{\gamma}_d$, and kurtosis $\bar{\kappa}_d$ of the accelerometer signals. The black error bars depict the standard error. The statistics are computed over a single axis of the raw acceleration signal, its first integration (velocity), and its second integration (position), as specified in the subcaptions. Integration was performed cumulatively via Simpson's rule over the complete signal before segmentation has taken place.

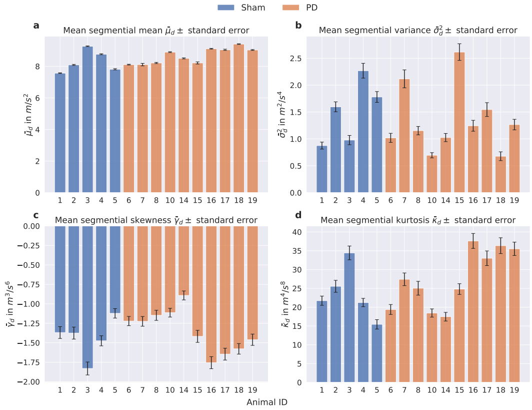

(a) z-axis

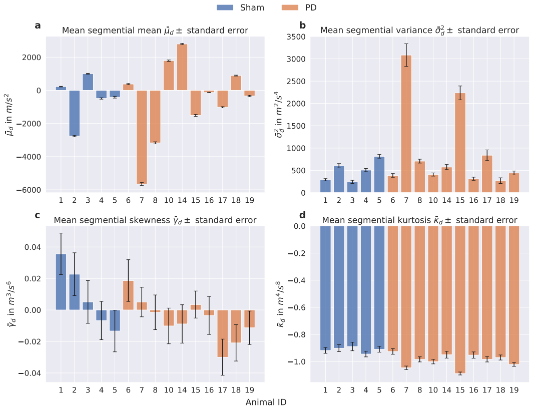

(b) z-axis, integrated once (velocity)

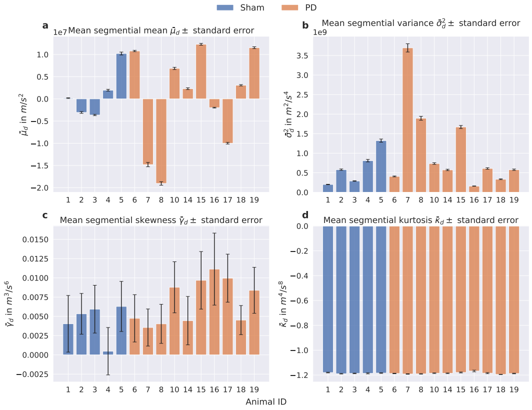

(c) z-axis, integrated twice (position)

Supplementary Figure S 3. Continuation of Figure 2

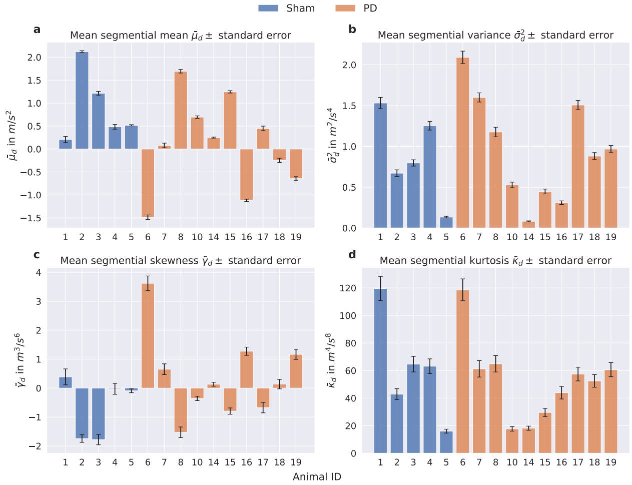

(a) ϕ-axis of spherical coordinates

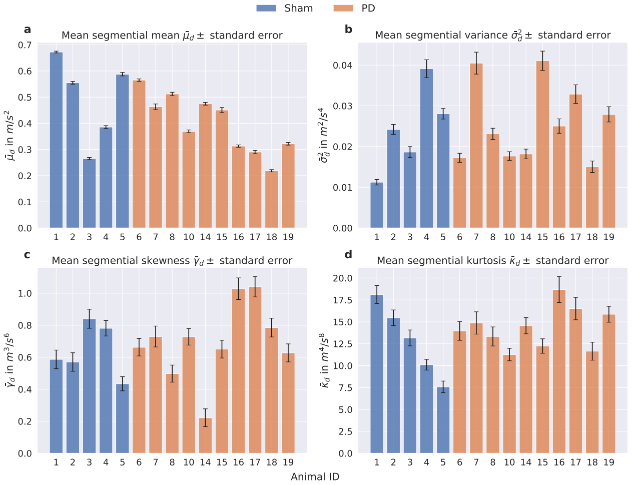

(b) θ-axis of spherical coordinates

Supplementary Figure S 4. Means of segmental mean $\bar{\mu}_d$, variance $\bar{\sigma}_d^2$, skewness $\bar{\gamma}_d$, and kurtosis $\bar{\kappa}_d$ of the accelerometer signals. The black error bars depict the standard error. The statistics are computed over the axes after transforming the 3D-acceleration signal into spherical coordinates.

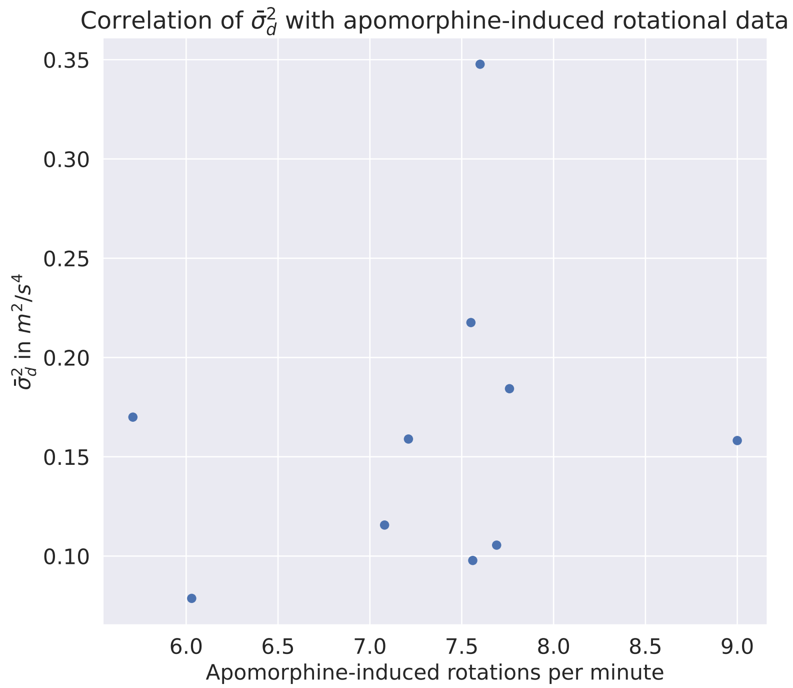

Supplementary Figure S 5. Apomorphine-induced rotations do not correlate with the mean segmental variance per dataset.
